# Polysaccharide-rich birch sap attenuates kainic acid-induced acute seizures by suppressing TLR4/NF-κB-mediated inflammation and preserving blood-brain barrier and glutamate homeostasis

**DOI:** 10.3389/fphar.2026.1885179

**Published:** 2026-07-22

**Authors:** Cheng-Wei Lu, Chia-Chan Wu, Kuan-Ming Chiu, Ming-Yi Lee, Wun-Jing Pan, Tzu-Yu Lin, Su-Jane Wang

**Affiliations:** 1 Department of Anesthesiology, Far-Eastern Memorial Hospital, New Taipei, Taiwan; 2 Department of Mechanical Engineering, Yuan Ze University, Taoyuan, Taiwan; 3 Division of Cardiovascular Surgery, Cardiovascular Center, Far-Eastern Memorial Hospital, New Taipei, Taiwan; 4 Department of Electrical Engineering, Yuan Ze University, Taoyuan, Taiwan; 5 Department of Medical Research, Far-Eastern Memorial Hospital, New Taipei, Taiwan; 6 Ph.D. Program in Pharmaceutical Biotechnology, College of Medicine, Fu-Jen Catholic University, New Taipei, Taiwan; 7 School of Medicine, Fu Jen Catholic University, New Taipei, Taiwan; 8 Research Center for Chinese Herbal Medicine, College of Human Ecology, Chang Gung University of Science and Technology, Taoyuan, Taiwan

**Keywords:** Birch sap polysaccharides, epilepsy, glutamate homeostasis, kainic acid, neuroprotection, TLR4/NF-kB signaling pathway

## Abstract

**Background:**

Plant polysaccharides possess significant anticonvulsant potential. This study investigated the neuroprotective effects of polysaccharide-rich birch sap (PRBS), previously characterized as primarily low-molecular-weight polysaccharides (MW·1.29 kDa), in a rat model of kainic acid (KA)-induced acute seizures.

**Methods:**

Rats received oral PRBS (6/10 mL/kg) for 7 days before KA-induced (15 mg/kg, i.p.) seizures. Seizure severity was evaluated using behavioral, electroencephalographic (EEG), and cerebral blood flow (CBF) monitoring. Brain tissues were analyzed via histology, Western blotting, and high-performance liquid chromatography (HPLC) for neuronal damage, neuroinflammation, blood-brain barrier (BBB) integrity, and glutamate metabolism.

**Results:**

Results demonstrated that the higher dose (10 mL/kg) exerted potent anticonvulsant effects, significantly reducing tonic convulsion scores and EEG ictal spikes. Furthermore, the treatment mitigated KA-induced CBF deficits and prevented neuronal damage in the cortex and hippocampus. Mechanistically, PRBS suppressed glial activation, indicated by a decrease in GFAP+ and OX42+ cells and reduced expression of markers associated with a pro-inflammatory state (C3 and CD86). It also inhibited neuroinflammatory signaling by downregulating TLR4 and p-IkB levels while suppressing the overall activation of NF-kB p65, thereby decreasing the release of pro-inflammatory cytokines (IL-1b, IL-6, and TNF-a). Additionally, the treatment attenuated BBB disruption by reducing albumin extravasation and upregulating tight junction proteins (claudin-5 and occludin). Finally, it restored glutamate homeostasis by upregulating the astrocytic transporter GLT-1 and glutamine synthetase, while downregulating glutamine transporters (SNAT1/3), glutaminase, and the vesicular glutamate transporter VGLUT1.

**Conclusion:**

PRBS exhibits potent antiseizure and neuroprotective properties that are closely associated with the modulation of TLR4/NF-kB-mediated inflammation, the preservation of BBB integrity, and the regulation of glutamate homeostasis, highlighting its potential as a natural therapeutic agent for seizures.

## 1 Introduction

Epilepsy is a chronic neurological disorder affecting approximately 70 million people worldwide (Feigin et al., 2019; Trinka et al., 2023). The disorder is characterized by recurrent spontaneous seizures arising from an imbalance between excitatory and inhibitory neurotransmission (Thijs et al., 2019; Löscher, 2021). These ictogenic events frequently trigger a pathological cascade; notably, the elevation of pro-inflammatory cytokines exacerbates neuroinflammation, which further drives neuronal hyperexcitability and excitotoxicity (Devinsky et al., 2013; Terrone et al., 2020; Wolinski et al., 2022). Despite the availability of numerous anti-seizure medications (ASMs), approximately 30% of patients remain refractory to current pharmacological interventions (Kalilani et al., 2018) Furthermore, ASMs are often associated with systemic side effects that can lead to treatment interruption or severely diminish quality of life (Akyüz et al., 2021). Consequently, the search for safe and efficacious antiepileptogenic agents—capable of preventing or modifying the disease process rather than merely suppressing symptoms—has become a primary focus of medical research. In this context, plant-derived compounds have emerged as a promising resource for the discovery of novel antiepileptogenic therapies (Dhir, 2020; Kumari et al., 2023; He et al., 2024).

Birch sap, a clear to light-yellow liquid extracted from the trunk of the *Betula* genus (family Betulaceae), is highly valued for its nutrient-dense profile. Rich in sugars, vitamins, minerals, and amino acids, it is widely utilized in the production of functional foods and drinks (Svanberg et al., 2012; Keita et al., 2021). Beyond its nutritional value, birch sap has traditionally served as a therapeutic agent for conditions such as hepatitis, skin rashes, intestinal parasites, and scurvy (Rastogi et al., 2015; Liu et al., 2024). Our recent findings demonstrated that birch sap is particularly enriched with low-molecular-weight polysaccharides (MW ≈ 1.29 kDa) and exhibits a strong safety profile alongside significant neuroprotective effects, specifically in mitigating memory impairment and enhancing cerebral blood flow (CBF) in rats (Huang C. F. et al., 2025). Its low molecular weight suggests better bioavailability and the potential to cross the blood-brain barrier (BBB). Given that plant-derived polysaccharides possess potent anti-inflammatory, antioxidant, and immunomodulatory properties (Zheng et al., 2020; Guo et al., 2023) and have emerged as promising anticonvulsant candidates (Li et al., 2022; Xie et al., 2022; Lu C. W. et al., 2024; Nonato et al., 2024), we hypothesized that the polysaccharide-rich birch sap (PRBS) may exert a modulatory role in epileptogenesis. To test this hypothesis, the present study aimed to: (i) evaluate the antiseizure effects of PRBS in a kainic acid (KA)-induced acute seizure rat model; (ii) benchmark its therapeutic potential against the conventional antiseizure medication, carbamazepine (CBZ); and (iii) elucidate the underlying molecular mechanisms, focusing on the modulation of neuroinflammation and glutamatergic hyperactivity−two pivotal drivers in the pathogenesis of epilepsy (Green et al., 2021; Soltani Khaboushan et al., 2022). To our knowledge, this is the first investigation to explore the antiseizure potential of PRBS.

## 2 Materials and methods

### 2.1 Animals

Male Sprague-Dawley (SD) rats (n = 60; 6–7 weeks old; weighing 180–200 g) were purchased from BioLASCO (Taipei, Taiwan). The animals were housed under standardized laboratory conditions, maintained at a temperature of 20 °C–23 °C and a relative humidity of 50%–60%, under a 12-h light/dark cycle. All rats had *ad libitum* access to standard laboratory chow and water. All experimental procedures were approved by the Institutional Animal Care and Use Committee (IACUC) of Fu Jen Catholic University (Approval No. A11146).

### 2.2 Birch sap and reagents

Birch sap was obtained from Forest Works (Hokkaido, Japan). The preparation and chemical characteristics of PRBS used in this study have been previously described (Huang C. F. et al., 2025). PRBS consists mainly of low-molecular-weight polysaccharides with an average molecular weight of approximately 1.29 kDa. Compositional analysis revealed that its most abundant monosaccharide is fructose, followed by glucose and fucose, in an approximate relative ratio of 8:5:3, respectively. This profile indicates that fructose is the most abundant monosaccharide in the extract, followed by glucose and fucose. All other chemical reagents, including KA (#K0205) and CBZ (#C4024), were purchased from Sigma–Aldrich (St. Louis, MO, United States).

### 2.3 Experimental design

**FIGURE 1:**
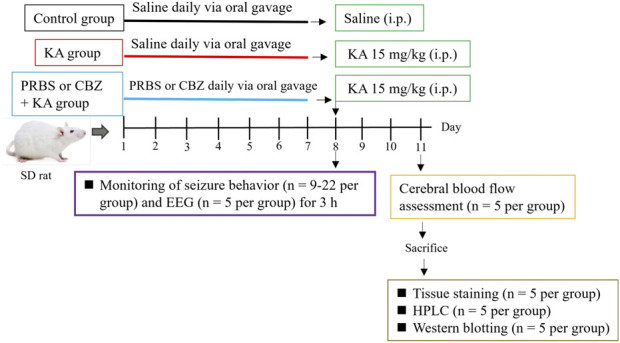
Schematic of the experimental design.

The KA-induced seizure model in rats exhibits pathological characteristics similar to those observed in patients with temporal lobe epilepsy (Rusina et al., 2021). The experimental design is illustrated in Figure 1. To prevent selection bias, an initial cohort of animals with matched body weights was numbered and randomly assigned to five groups by an independent, blinded researcher. These groups were: Control, KA, PRBS (6 mL/kg) + KA, PRBS (10 mL/kg) + KA, and CBZ (100 mg/kg) + KA. To evaluate the protective effects of PRBS against KA-induced acute seizures, rats received a daily oral gavage of normal saline, PRBS, or CBZ for seven consecutive days. On day 8, seizures were induced via an intraperitoneal (i.p.) injection of KA (15 mg/kg) was administered to induce seizures. Behavioral seizures and electroencephalograms (EEGs) were recorded for 3 h post-injection. Seizure onset latency (min) was measured, and seizure severity was scored using the Racine scale (Racine, 1972). To ensure model reliability, predefined exclusion criteria were applied; rats failing to reach at least stage IV on the Racine scale or presenting with unreadable EEG recordings were excluded. Additionally, four rats (∼10%) died from acute KA-induced continuous seizures. After accounting for these exclusions and mortalities, the final sample size was 10–12 rats per group for all downstream evaluations.

On day 11 (72 h post-KA injection), cerebral blood flow (CBF) was measured. Subsequently, the rats were euthanized for tissue staining, high-performance liquid chromatography (HPLC), and Western blot analyses. Dosages and administration schedules were determined based on previous literature and pilot studies (Chang et al., 2022; Hung et al., 2024; Huang C. F. et al., 2025). This approach aimed to minimize animal use in accordance with ethical guidelines while maintaining sufficient statistical power to detect significant differences between groups. CBZ was utilized as a positive control. The 100 mg/kg dose was selected based on the FDA guidelines for body surface area normalization; it translates to a human equivalent dose of approximately 16.1 mg/kg (equivalent to ∼960 mg/day for a 60-kg adult), aligning with the standard clinical therapeutic range (Nair and Jacob, 2016).

### 2.4 EEG recording

Following established protocols (Jean et al., 2022; Lu et al., 2025), global cortical activity was recorded using a three-channel EEG acquisition system (Pinnacle Technology, Lawrence, KS, United States). Briefly, rats (n = 5 per group) were anesthetized with 3% isoflurane (RWD Life Science, Shenzhen, China) and secured in a stereotaxic frame. To capture widespread EEG dynamics, four electrodes were surgically implanted into the frontal (AP +1.8 mm, ML ±1.5 mm) and parietal (AP −2.9 mm, ML ±2.5 mm) cortices relative to bregma, based on the Paxinos and Watson rat brain atlas. Following a 3-day recovery period, the rats were placed in transparent observation chambers and connected to an amplifier (model 8213-SE3; Pinnacle Technology). Continuous EEG recordings were acquired for 3 h post-KA injection. Electrographic seizures were defined as generalized, rhythmic spike or spike-and-wave discharges with an amplitude >60 μV and a duration >10 s. Post-acquisition analysis of ictal spike frequency and duration were conducted using PAL-8200 software (Pinnacle Technology).

### 2.5 CBF analysis

CBF was monitored in real-time using a laser speckle imaging system (RFLSI III; RWD Life Science, Shenzhen, China), as previously described (Huang C. F. et al., 2025). Following anesthesia induction with 3% isoflurane, the rats (n = 5 per group) were secured in a stereotaxic apparatus. The skull was exposed via a midline scalp incision and maintained dry to ensure optimal imaging quality. Blood flow speckle patterns were recorded using a high-resolution CMOS camera under laser illumination. Data were acquired for 15 s at a working distance of 25 cm, covering a 1.7-cm field of view with a resolution of 2064 × 1,544 pixels. Raw blood flow intensity was initially quantified in perfusion units (PU). To evaluate relative perfusion changes, these raw data were converted to baseline-normalized CBF, expressed as a ratio to the pre-intervention baseline.

### 2.6 Brain tissue staining

Rats (n = 5 per group) were deeply anesthetized with 3% isoflurane and transcardially perfused with 100 mL of 0.9% saline, followed by 50 mL of 4% paraformaldehyde (PFA) in 100 mM phosphate-buffered saline (PBS; pH 7.4). Brains were harvested, post-fixed in the same fixative at 4 °C for 24 h, and cryoprotected in 30% sucrose in PBS at 4 °C for 7 days until saturated. Coronal sections (30 μm thick) were sectioned using a freezing microtome and mounted on gelatin-coated slides.

Nissl staining was performed to assess neuronal loss and morphological changes. Briefly, sections were rehydrated and stained with 0.5% cresyl violet (#ab246816, Abcam, Cambridge, United Kingdom) for 6 min. For the detection of degenerating neurons, Fluoro-Jade B (FJB) staining was performed as previously described (Jean et al., 2022; Hung et al., 2024). Sections were pretreated with 0.06% potassium permanganate for 20 min and incubated in 0.0004% FJB (Biosensis, Thebarton, South Australia) for 40 min in the dark.

To evaluate astrocytic and microglial activation, sections underwent immunohistochemistry for glial fibrillary acidic protein (GFAP) and OX42, respectively (Hung et al., 2024; Lu et al., 2025). Following a 30-min block in 5% normal horse serum, sections were incubated overnight at 4 °C with primary antibodies against GFAP (1:500; Cell Signaling, MA, United States) or OX42 (1:500; Bio-Rad, Hercules, CA, United States). Sections were then incubated with a biotinylated secondary antibody (1:200; GeneTex, MI, United States) for 2 h, followed by ExtrAvidin–peroxidase (1:1,000) for 1 h. Immunoreactivity was visualized using a 3,3′-diaminobenzidine (DAB) kit (Vector Laboratories, Burlingame, CA, United States). Following their respective staining protocols, all slides were dehydrated in graded ethanol, cleared in xylene, and coverslipped with dibutylphthalate polystyrene xylene (DPX, Sigma-Aldrich, St. Louis, MO, United States).

Images of the entorhinal cortex and hippocampal CA1/CA3 regions were captured at ×100 magnification using a Zeiss Axioskop 40 microscope. To determine cell density (cells/mm^2^), a fixed rectangular region of interest (ROI, 250 × 250 μm) was applied to each captured area. Cells were quantified manually across four consecutive sections per animal using the Multi-point tool in ImageJ software (NIH, Bethesda, MD, United States). Slides were randomly coded by an independent investigator. Cell counting was performed blind, and unblinding occurred post-analysis.

### 2.7 Brain tissue collection for HPLC and Western blot analysis

Rats (n = 5 per group) were euthanized via decapitation. The brains were immediately removed, and the cortex and hippocampus were dissected on ice. The tissues were then snap-frozen and stored at −80 °C until further analysis.

### 2.8 HPLC analysis

Glutamate and γ-aminobutyric acid (GABA) concentrations within the cortex and hippocampus were quantified via HPLC following established protocols (Hung et al., 2024; Lu et al., 2025). Briefly, 50 mg of brain tissue was homogenized in PBS and centrifuged at 15,000 × g for 10 min at 4 °C. The resulting supernatant (10 μL) was passed through a 0.22-µm membrane filter before analysis using an HTEC-500 system (Eicom, Kyoto, Japan). Separation was achieved using an Eicompak FA-3ODS reverse-phase column (3.0 mm × 75 mm) equipped with a CA-ODS precolumn (3.0 mm × 4.0 mm). The mobile phase consisted of 0.1 M phosphate buffer (pH 6.0, containing 0.05 M sodium sulfate, 5 mg/L EDTA, 13% acetonitrile, and 7% methanol). Detection was performed using a glassy carbon working electrode (WE-GC) set at +600 mV vs. Ag/AgCl. With the column temperature maintained at 40 °C and a flow rate of 0.5 mL/min, glutamate and GABA levels were calculated based on peak areas relative to external standards. Data are expressed as ng/mg protein.

### 2.9 Western blot analysis

Frozen hippocampal and cortical tissues were homogenized via ultrasonication in a lysis buffer containing 2% SDS, 1 mM PMSF, 1 mM Na_2_VO_4_, and 20 mM NaF, supplemented with a protease and phosphatase inhibitor cocktail (Sigma-Aldrich, St. Louis, MO, United States). The homogenates were heated at 95 °C for 10 min to denature proteins, followed by centrifugation at 10,000 × g (10 min, 4 °C) to pellet cellular debris. The Bradford assay (Bio-Rad Laboratories, Hercules, CA, United States) was employed to determine protein concentrations in the resulting supernatants. Equal protein loads (20 μg/lane) were resolved using 10% SDS-PAGE and electroblotted onto PVDF membranes (Thermo Fisher Scientific, Waltham, MA, United States). To prevent non-specific binding, membranes were blocked for 40 min at room temperature using 5% bovine serum albumin in TBST (TBS with 0.1% Tween-20). Membranes were then incubated overnight at 4 °C with the primary antibodies listed in Table 1. After 5-min washes in TBST, membranes were incubated with HRP-conjugated secondary antibodies (1:2,000; GeneTex, Irvine, CA, United States) for 2 h at 25 °C. Protein bands were visualized using enhanced chemiluminescence (ECL) reagents (Amersham Biosciences, United Kingdom). The resulting signals were scanned and densitometrically analyzed using ImageJ software (NIH, Bethesda, MD, United States). Target protein expression was normalized to β-actin. The stability of β-actin as a loading control was validated by comparing its raw densitometry values across all experimental treatments, which showed no significant variation, ensuring that it was not affected by the experimental interventions.

**TABLE 1:** List of primary antibodies.

Target protein	Dilution	Manufacturer
IL-1β (ab283818), IL-6 (ab9324), TNF-α (ab6671), C3 (ab200999), GLT-1 (ab41621), GS (ab176562), VGLUT1 (ab134283), TLR4 (ab13556), Occiudin (ab216327)	1:300-1:50,000	Abcam
Claudin5 (ab131259)		
CD86 (GTX34569)	1:1,000	Gene Tex
p-IκB (#9246), p-NFκB (#3033), albumin (#4929)	1:500-1:1,000	Cell signaling
SNAT3 (14315-1-AP)	1:800	Proteintech
SNAT1 (ANT-184)	1:800	Alomone labs
Glutaminase (701,965)	1:10,000	Thermo Fisher

### 2.10 Statistical analysis

Sample size adequacy was confirmed *post hoc* using G*Power software (version 3.1). A sensitivity power analysis indicated that the final analyzed sample size (Total N = 60, n = 10–12 per group across 5 groups) was sufficient to detect a minimum effect size of Cohen’s f = 0.55 for a one-way ANOVA, assuming an alpha level of 0.05% and 80% statistical power. Data are presented as the mean ± standard error of the mean (S.E.M.), with individual data points superimposed on bar graphs to visualize biological variability. All statistical analyses were performed using GraphPad Prism version 8.1 (GraphPad Software, San Diego, CA, United States). The Kolmogorov–Smirnov and Brown–Forsythe tests were used to confirm data normality and homogeneity of variance, respectively. Multiple group comparisons were evaluated using a one-way analysis of variance (ANOVA) followed by Tukey’s *post hoc* test. To comprehensively assess the magnitude and precision of the findings, effect sizes (expressed as *R* ^2^) and 95% confidence intervals (CIs) of the mean differences were calculated for all key analyses. Statistical significance was defined as *p* < 0.05.

## 3 Results

### 3.1 PRBS exerts a potent preventive effect on KA-induced acute seizure activity

To evaluate the anticonvulsant potential of PRBS, rats received either PRBS (6 or 10 mL/kg) or CBZ (100 mg/kg) via daily oral gavage for seven consecutive days prior to seizure induction with KA (15 mg/kg, i.p.). Behavioral assessments revealed that pretreatment with 10 mL/kg PRBS or CBZ significantly prolonged seizure latency and attenuated seizure severity compared to the KA group [F (3,60) = 40.43, *p* < 0.0001, *R* ^2^ = 0.60; Table 2]. Conversely, the lower dose of PRBS (6 mL/kg) produced no significant effect on seizure scores compared to the KA group (*p* = 0.89). The seizure incidence in the KA group reached 91% (20/22), but was markedly reduced to 14% (3/22) and 10% (1/11) in the 10 mL/kg PRBS and CBZ groups, respectively. Notably, the anticonvulsant efficacy of the 10 mL/kg dose was significantly greater than that of the 6 mL/kg dose (*p* < 0.0001) and statistically comparable to that of the positive control, CBZ (*p* = 0.92); consequently, this higher dose was selected for subsequent mechanistic studies.

**TABLE 2:** Effect of PRBS pretreatment on KA-induced seizures in rats.

Parameters	KA	PRBS 6 mL/kg + KA	PRBS 10 mL/kg + KA	CBZ 100 mg/kg + KA
Seizure score	4.5 ± 0.1	4.1 ± 0.3	1.2 ± 0.5***	0.9 ± 0.5***
Latency to first seizure (min)	83.8 ± 6.5	108.8 ± 8.6	165.5 ± 14.5***	140^a^
% seizure	91% (20/22)	66% (6/9)	14% (3/22)	10% (1/11)

***p < 0.0001 vs. KA, group. a: Only 1 out of 11 animals in the CBZ, group exhibited seizures; therefore, the latency value represents a single measurement (n = 1).

**FIGURE 2:**
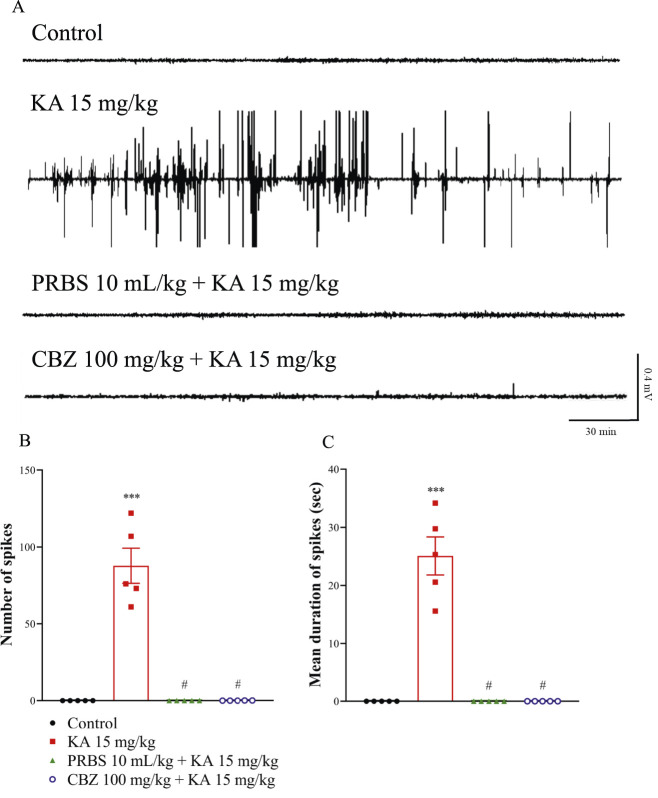
Effect of PRBS on EEG activity in rats. **(A)** Representative EEG traces recorded from the cortex in rats. **(B,C)** Number and duration of seizure spikes. The number and duration of spikes were assessed for 3 h after KA injection. Individual data points are plotted to illustrate the distribution of biological replicates. Statistical significance was determined using one-way ANOVA followed by Tukey’s *post hoc* test. ***p < 0.0001 vs. control group; #*p* < 0.05 vs. KA group (n = 5 per group).

To corroborate the behavioral findings, global EEG analysis was performed to quantify generalized ictal spikes across the cortex. The KA group exhibited a significant increase in both spike number [F (3,16) = 56.93, *p* < 0.0001, *R* ^2^ = 0.92; 95% CI of increase: 64.67 to 110.90] and duration [F (3,16) = 58.55, *p* < 0.0001, *R* ^2^ = 0.92; 95% CI of increase: 18.45 to 31.71] compared to the control group (Figures 2A–C). In contrast, pretreatment with either PRBS (10 mL/kg) or CBZ (100 mg/kg) significantly attenuated these KA-induced electrographic abnormalities, showing a marked reduction in both metrics (*p* < 0.0001 vs. KA group; n = 5). Both the behavioral and EEG profiles of the PRBS group were statistically indistinguishable from those of the CBZ group (*p* = 1.00), demonstrating that PRBS possesses an anticonvulsant potency comparable to that of the standard antiepileptic drug CBZ.

### 3.2 PRBS ameliorates KA-induced deficits in CBF

**FIGURE 3:**
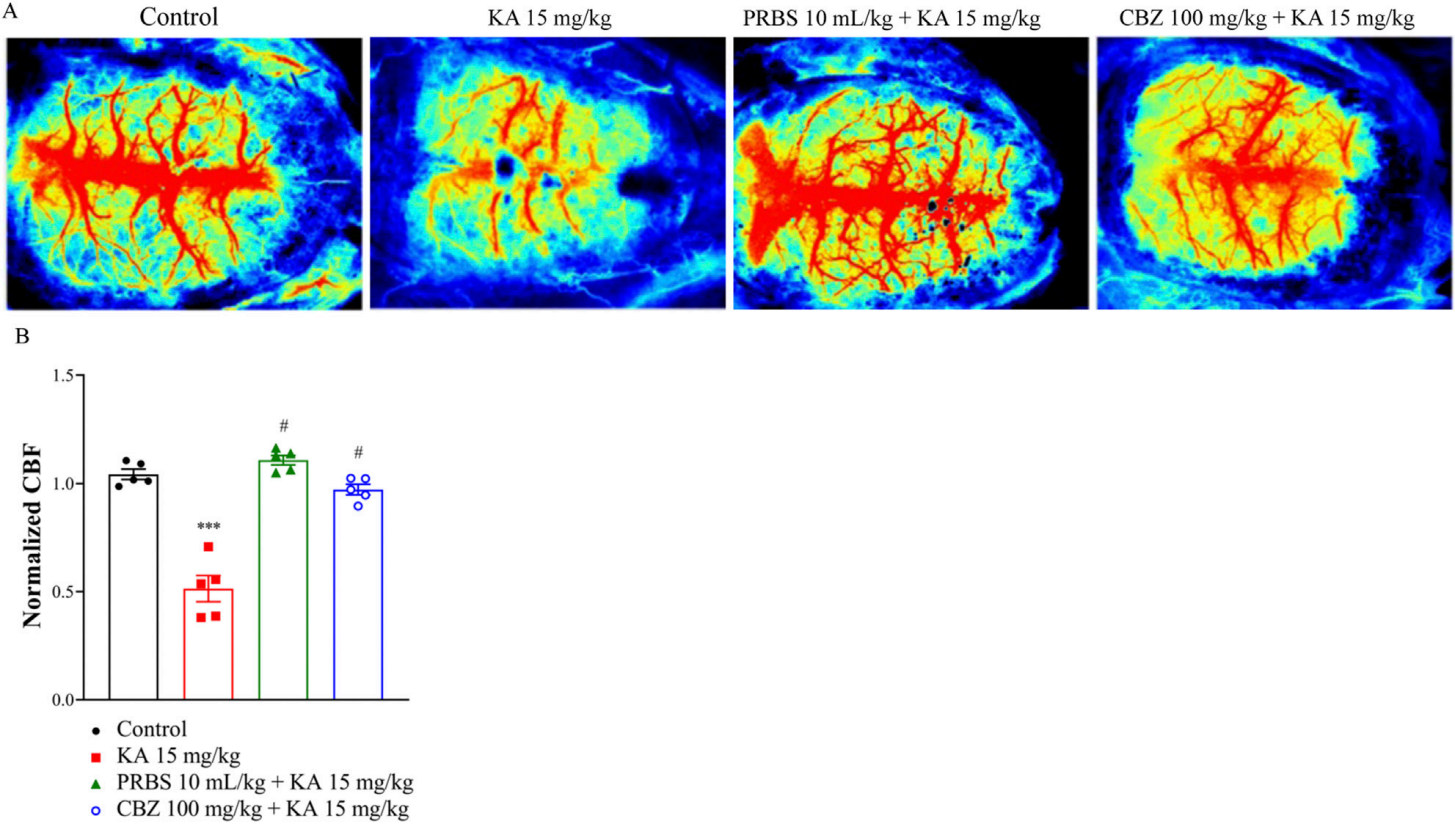
Effect of PRBS on CBF in rats. **(A)** Representative images of CBF across experimental groups. **(B)** Quantification of baseline-normalized CBF (expressed as a ratio to baseline). Individual data points are plotted to illustrate the distribution of biological replicates. Statistical significance was determined using one-way ANOVA followed by Tukey’s *post hoc* test. ***p < 0.0001 vs. control group; #*p* < 0.001 vs. KA group (n = 5 per group).

Because changes in CBF are closely associated with KA-induced acute seizures (Lu C. W. et al., 2024; Lu et al., 2025), we assessed baseline-normalized CBF across all experimental groups. The KA group exhibited a significant reduction in normalized CBF compared to the control group [F (3,16) = 54.4, *p* < 0.0001, *R* ^2^ = 0.91; 95% CI of reduction: 0.38 to 0.61; Figures 3A,B], indicating substantial cerebrovascular impairment. Pretreatment with PRBS (10 mL/kg) or CBZ (100 mg/kg) effectively mitigated this decline, maintaining normalized CBF near pre-intervention baseline levels (*p* < 0.0001 vs. KA group). Furthermore, no statistically significant difference was observed between the PRBS and CBZ treatment groups (*p* = 0.13). These results suggest that CBF stabilization is closely associated with the neuroprotective effects of PRBS in this seizure model.

### 3.3 PRBS attenuates KA-induced neuronal damage in the entorhinal cortex and hippocampus

**FIGURE 4:**
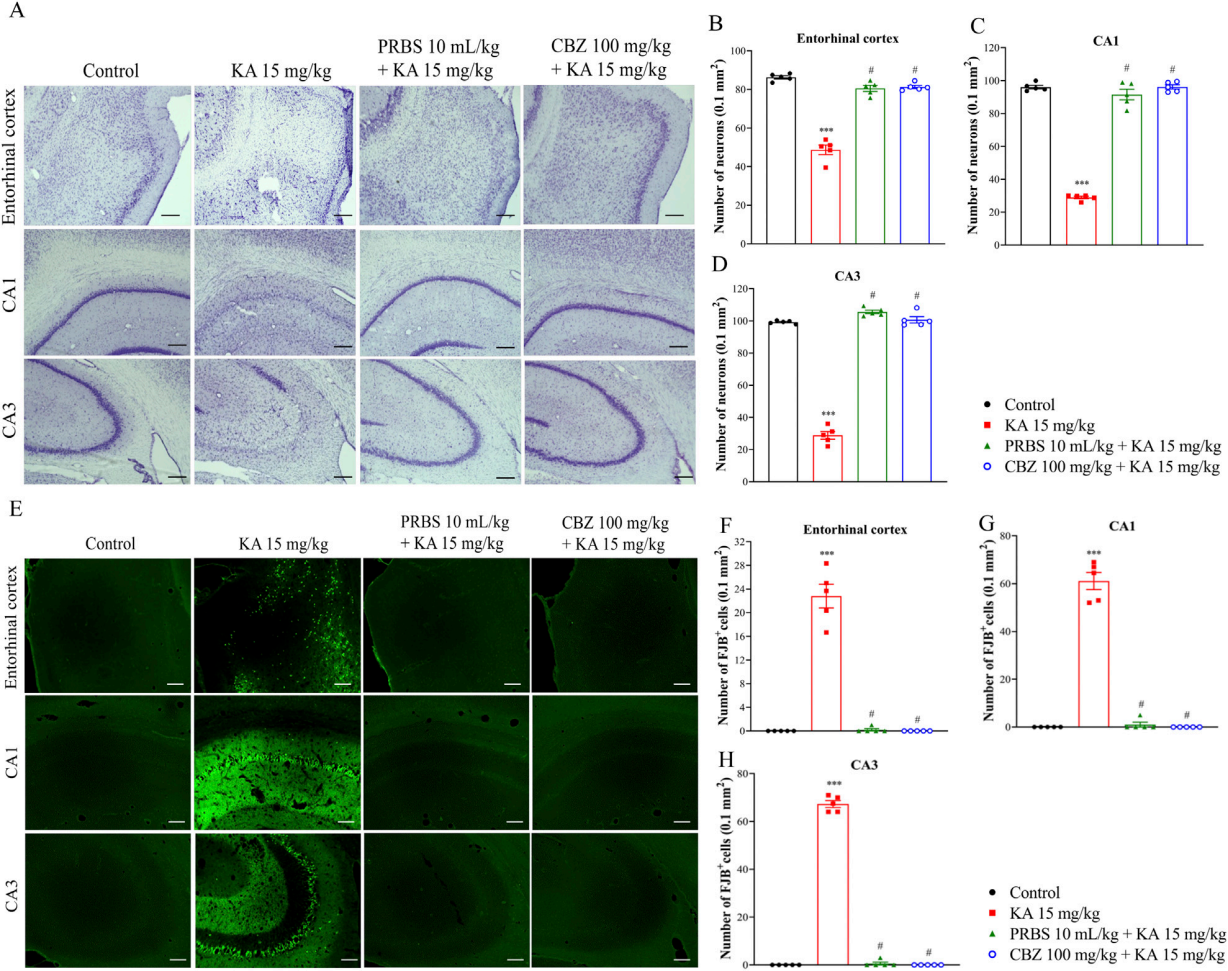
Effect of PRBS on the structural integrity and neurodegeneration of the entorhinal cortex and hippocampus (CA1 and CA3). **(A, E)** Representative images of cresyl violet and FJB staining in the rat entorhinal cortex and hippocampus. **(B−D, F−H)** Quantification of labeled cells in the entorhinal cortex and hippocampus across different experimental groups. Individual data points are plotted to illustrate the distribution of biological replicates. Statistical significance was determined using one-way ANOVA followed by Tukey’s *post hoc* test. ***p < 0.0001 vs. control group; #*p* < 0.05 vs. KA group (n = 5 per group).

To examine whether PRBS prevents neuronal damage in KA-injected brains, histological assessments using Nissl staining and FJB labeling were performed to evaluate structural integrity and neurodegeneration, respectively. Nissl-stained sections from the control group displayed densely packed neurons with intact morphology and a well-organized laminar distribution in the entorhinal cortex and hippocampal subfields (CA1 and CA3) (Figure 4A). KA administration severely disrupted these cellular arrangements and induced profound neuronal loss in these regions compared to the control group [Entorhinal cortex: F (3,16) = 118.2, *p* < 0.0001, *R* ^2^ = 0.95; 95% CI of decrease: 31.9 to 43.94; CA1: F (3,16) = 310.6, *p* < 0.0001, *R* ^2^ = 0.98; 95% CI of decrease: 59.6 to 74.71; CA3: F (3,16) = 496.00, *p* < 0.0001, *R* ^2^ = 0.98; 95% CI of decrease: 63.72 to 77.01; Figure 4B−D]. Pretreatment with either PRBS or CBZ effectively preserved the cytoarchitecture and significantly attenuated this reduction in neuronal density (n = 5; *p* < 0.0001 vs. KA group). Furthermore, while FJB-positive signals were absent in control rats, KA administration triggered extensive neurodegeneration, evidenced by a dramatic increase in FJB-positive neurons [Entorhinal cortex: F (3,16) = 128.1, *p* < 0.0001, *R* ^2^ = 0.96; 95% CI of increase: 18.70 to 26.80; CA1: F (3,16) = 266.5, *p* < 0.0001, *R* ^2^ = 0.98; 95% CI of increase: 53.50 to 68.60; CA3: F (3,16) = 180.1, *p* < 0.0001, *R* ^2^ = 0.99; 95% CI of increase: 64.10 to 70.50; Figures 4E–H]. This degenerative effect was nearly abolished by pretreatment with PRBS or CBZ, resulting in significantly fewer FJB-positive cells relative to the KA group (n = 5; *p* < 0.0001 vs. KA group). Consistent with the behavioral and EEG data, the degree of neuroprotection did not differ significantly between the PRBS and CBZ groups [Entorhinal cortex; *p* = 0.99; CA1: *p* = 0.98; CA3: *p* = 0.95].

### 3.4 PRBS suppresses KA-induced glial activation and the elevation of pro-inflammatory cytokines in the cortex and hippocampus

**FIGURE 5:**
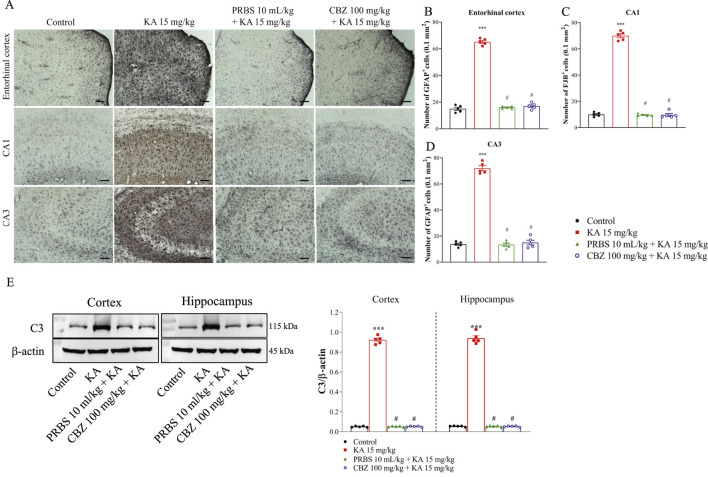
Effect of PRBS on astrocyte activation in the entorhinal cortex and hippocampus. **(A)** Representative immunostaining images of GFAP-positive astrocytes in the entorhinal cortex and hippocampal CA1 and CA3 regions. **(B–D)** Quantitative analysis of GFAP-positive cells in these regions. **(E)** Representative Western blots and densitometric analysis of the reactive astrocyte marker C3 in the cortex and hippocampus. Individual data points are plotted to illustrate the distribution of biological replicates. Statistical significance was determined using one-way ANOVA followed by Tukey’s *post hoc* test. ***p < 0.0001 vs. control group; #*p* < 0.05 vs. KA group (n = 5 per group).

**FIGURE 6:**
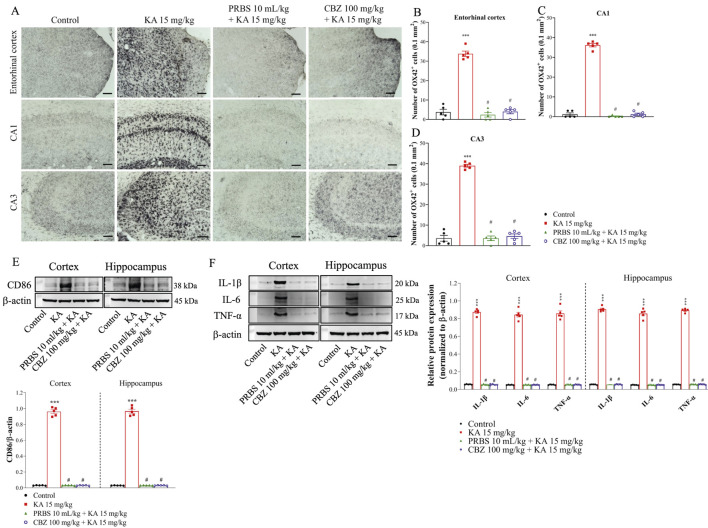
Effect of PRBS on microglial activation in the entorhinal cortex and hippocampus. **(A)** Representative immunostaining images of OX42-positive microglia in the entorhinal cortex and hippocampal CA1 and CA3 regions. **(B–D)** Quantitative analysis of OX42-positive cells in these regions. **(E,F)** Representative immunoblots and corresponding densitometric analyses of **(E)** the reactive microglial marker CD86, and **(F)** pro-inflammatory cytokines IL-1β, IL-6, and TNF-α in the cortex and hippocampus. Individual data points are plotted to illustrate the distribution of biological replicates. Statistical significance was determined using one-way ANOVA followed by Tukey’s *post hoc* test. ***p < 0.0001 vs. control group; #*p* < 0.05 vs. KA group (n = 5 per group).

Glial cell activation plays a critical role in neuronal injury during seizures (Devinsky et al., 2013; Vezzani et al., 2013). Immunohistochemical staining revealed that the numbers of GFAP-positive astrocytes and OX42-positive microglia were significantly elevated in the entorhinal cortex and hippocampus of the KA group compared to controls [GFAP: Entorhinal cortex, F (3,16) = 665.7, *p* < 0.0001, *R* ^2^ = 0.99; 95% CI of increase: 46.3 to 54.0; CA1: F (3,16) = 860.7, *p* < 0.0001, *R* ^2^ = 0.99; 95% CI of increase: 55.84 to 64.10; CA3: F (3,16) = 365, *p* < 0.0001, *R* ^2^ = 0.98; 95% CI of increase: −64.34 to −52, Figures 5A–D; OX42: Entorhinal cortex, F (3,16) = 100.2, *p* < 0.0001, *R* ^2^ = 0.94; 95% CI of increase: 23.90 to 36.0; CA1: F (3,16) = 165.3, *p* < 0.0001, *R* ^2^ = 0.96; 95% CI of increase: 29.5 to 40.4; CA3: F (3,16) = 178.5, *p* < 0.0001, *R* ^2^ = 0.97; 95% CI of increase: 30.13 to 40.67, Figures 6A–D]. PRBS and CBZ treatments significantly attenuated this glial activation (*p* < 0.0001 vs. KA group). These histological findings were corroborated by Western blot analysis, which showed that the protein expression of C3 (a reactive astrocyte marker) and CD86 (a reactive microglia marker) was significantly elevated in the cortex and hippocampus of the KA group [C3: Cortex, F (3,16) = 2,394, *p* < 0.0001, *R* ^2^ = 0.99; 95% CI of increase: 0.83 to 0.91; Hippocampus: F (3,16) = 1,278, *p* < 0.0001, *R* ^2^ = 0.99; 95% CI of increase: 0.83 to 0.93, Figure 5E; CD86: Cortex, F (3,16) = 1,504, *p* < 0.0001, *R* ^2^ = 0.99; 95% CI of increase: 0.88 to 0.98; Hippocampus: F (3,16) = 1,399, *p* < 0.0001, *R* ^2^ = 0.99; 95% CI of increase: 0.89 to 0.99, Figure 6E]. PRBS or CBZ treatment effectively decreased these protein levels (*p* < 0.0001 vs. KA group), with PRBS demonstrating an inhibitory efficacy comparable to CBZ (cortex; *p* = 1.00; Hippocampus: *p* = 1.00).

Because pro-inflammatory reactive astrocytes and microglia secrete cytokines that exacerbate seizure-induced neuronal injury (Liu et al., 2025), we quantified IL-1β, IL-6, and TNF-α expression in the cortex and hippocampus via Western blot. KA administration triggered a profound upregulation of these cytokines compared to the control group [IL-1β: Cortex, F (3,16) = 2,727, *p* < 0.0001, *R* ^2^ = 0.99; 95% CI of increase: 0.78 to 0.84; Hippocampus: F (3,16) = 4,430, *p* < 0.0001, *R* ^2^ = 0.99; 95% CI of increase: 0.83 to 0.87; IL-6: Cortex, F (3,16) = 901.3, *p* < 0.0001, *R* ^2^ = 0.99; 95% CI of increase: 0.74 to 0.84; Hippocampus: F (3,16) = 1,205, *p* < 0.0001, *R* ^2^ = 0.99; 95% CI of increase: 0.75 to 0.85; TNF-α: Cortex, F (3,16) = 713, *p* < 0.0001, *R* ^2^ = 0.99; 95% CI of increase: 0.74 to 0.86; Hippocampus: F (3,16) = 7,894, *p* < 0.0001, *R* ^2^ = 0.99; 95% CI of increase: 0.81 to 0.85; Figure 6F]. Notably, these pathological elevations were significantly and equally attenuated by both PRBS and CBZ interventions (*p* < 0.0001 vs. KA group; *p* = 1.00 between treatments).

### 3.5 PRBS inhibits KA-induced TLR4/NF-κB activation in the cortex and hippocampus

**FIGURE 7:**
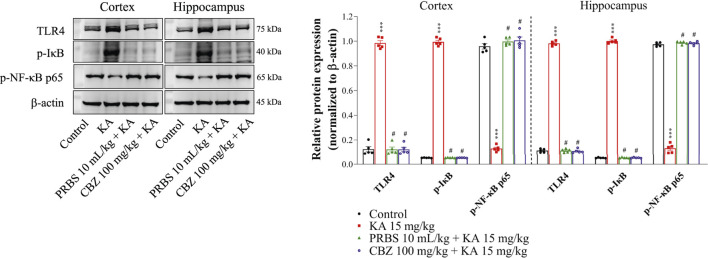
Effect of PRBS on the expression of TLR4, p-IκB, and cytosolic p-NF-κB p65 in the cortex and hippocampus. Representative immunoblots and quantitative densitometric analyses of TLR4, p-IκB, and cytosolic p-NF-κB p65 in the indicated regions are shown. Individual data points are plotted to illustrate the distribution of biological replicates. Statistical significance was determined using one-way ANOVA followed by Tukey’s *post hoc* test. ***p < 0.0001 vs. control group; #*p* < 0.05 vs. KA group (n = 5 per group).

Activation of the TLR4/NF-κB signaling pathway drives the neuroinflammatory responses crucial for epileptogenesis (Yue et al., 2023). Western blot analysis revealed that KA treatment markedly elevated TLR4 and p-IκB levels in the cortex and hippocampus, while significantly reducing cytosolic phosphorylated NF-κB p65 (p-NF-κB p65) levels compared to the control group [TLR4: Cortex, F (3,16) = 476.2, *p* < 0.0001, *R* ^2^ = 0.98; 95% CI of increase: 0.78 to 0.94; Hippocampus: F (3,16) = 3,707, *p* < 0.0001, *R* ^2^ = 0.99; 95% CI of increase: 0.84 to 0.89; p-IκB: Cortex, F (3,16) = 4,811, *p* < 0.0001, *R* ^2^ = 0.99; 95% CI of increase: 0.91 to 0.96; Hippocampus: F (3,16) = 10,582, *p* < 0.0001, *R* ^2^ = 0.99; 95% CI of increase: 0.93 to 0.96; p-NF-κB p65: Cortex, F (3,16) = 463.2, *p* < 0.0001, *R* ^2^ = 0.98; 95% CI of decrease: 0.74 to 0.90; Hippocampus: F (3,16) = 1912, *p* < 0.0001, *R* ^2^ = 0.99; 95% CI of decrease: 0.80 to 0.88; Figure 7]. Coupled with the marked elevation of downstream pro-inflammatory cytokines (as described above), this reduction in the cytosolic pool is consistent with the overall activation of the NF-κB signaling cascade. Conversely, pretreatment with PRBS or CBZ significantly attenuated the upregulation of TLR4 and p-IκB and restored cytosolic p-NF-κB p65 levels (*p* < 0.0001 vs. KA group), supporting an effective suppression of this inflammatory axis.

### 3.6 PRBS attenuates KA-induced albumin extravasation and tight junction protein downregulation in the cortex and hippocampus

**FIGURE 8:**
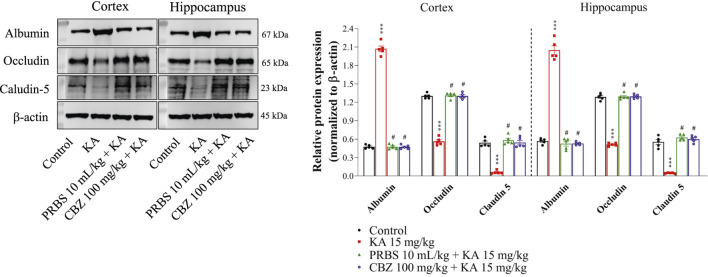
Effect of PRBS on the expression of albumin, claudin-5, and occludin in the cortex and hippocampus. Representative immunoblots and quantitative densitometric analyses of albumin, claudin-5, and occludin in the indicated regions are shown. Individual data points are plotted to illustrate the distribution of biological replicates. Statistical significance was determined using one-way ANOVA followed by Tukey’s *post hoc* test. ***p < 0.0001 vs. control group; #*p* < 0.05 vs. KA group (n = 5 per group).

Reactive gliosis and neuroinflammation are key contributors to blood-brain barrier (BBB) disruption in epilepsy (Huang N. et al., 2025; Onat et al., 2025). To evaluate BBB integrity, we quantified the expression of albumin—a marker of vascular extravasation—and the tight junction proteins claudin-5 and occludin. KA treatment markedly elevated albumin levels while downregulating claudin-5 and occludin compared to the control group [Albumin: Cortex, F (3,16) = 902, *p* < 0.0001, *R* ^2^ = 0.99; 95% CI of increase: 1.48 to 1.70; Hippocampus: F (3,16) = 315.8, p < 0.0001, *R* ^2^ = 0.98; 95% CI of increase: 1.30 to 1.65; Claudin-5: Cortex, F (3,16) = 74.69, *p* < 0.0001, *R* ^2^ = 0.93; 95% CI of decrease: 0.36 to 0.59; Hippocampus: F (3,16) = 116.3, *p* < 0.0001, *R* ^2^ = 0.95; 95% CI of decrease: 0.40 to 0.60; Occludin: Cortex, F (3,16) = 274, p < 0.0001, *R* ^2^ = 0.98; 95% CI of decrease: 0.64 to 0.82; Hippocampus: F (3,16) = 470.3, *p* < 0.0001, *R* ^2^ = 0.98; 95% CI of decrease: 0.70 to 0.84; Figure 8], indicating pronounced BBB compromise. Pretreatment with PRBS or CBZ effectively mitigated these pathological changes, suppressing albumin leakage and restoring tight junction protein expression (*p* < 0.0001 vs. KA group), with comparable efficacy between the two treatments (albumin and occludin, *p* = 1.00; claudin-5, *p* = 0.80).

### 3.7 PRBS prevents KA-induced alterations in glutamate levels in the cortex and hippocampus

**FIGURE 9:**
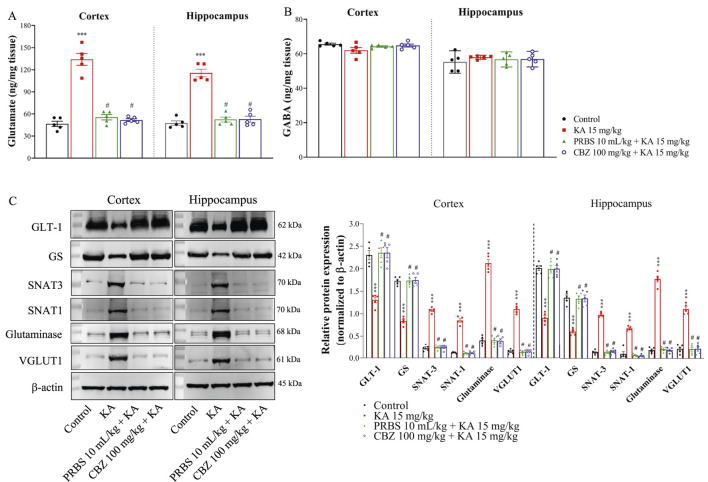
Effect of PRBS on glutamate and GABA concentrations, and protein expression of GLT-1, GS, SNAT1/3, glutaminase, and VGLUT1 in the cortex and hippocampus. **(A,B)** Glutamate and GABA concentrations in cortical and hippocampal brain tissue. **(C)** Representative immunoblots and corresponding densitometric analyses of GLT-1, GS, SNAT1/3, glutaminase, and VGLUT1 in the indicated regions are shown. Individual data points are plotted to illustrate the distribution of biological replicates. Statistical significance was determined using one-way ANOVA followed by Tukey’s *post hoc* test. ***p < 0.0001 vs. control group; #*p* < 0.05 vs. KA group (n = 5 per group).

An imbalance between excitatory (glutamate) and inhibitory (GABA) neurotransmission is a hallmark of epileptogenesis (Löscher, 2021). HPLC analysis revealed that KA administration triggered a significant elevation in glutamate levels within both the cortex and hippocampus compared to the control group [Cortex, F (3,16) = 73.31, *p* < 0.0001, *R* ^2^ = 0.93; 95% CI of increase: 67.80 to 107.10; Hippocampus: F (3,16) = 66.7, *p* < 0.0001, *R* ^2^ = 0.93; 95% CI of increase: 51.80 to 83.90; Figure 9A]. Pretreatment with either PRBS or CBZ effectively neutralized this surge (*p* < 0.0001 vs. KA group), maintaining glutamate at levels statistically indistinguishable from the control group (n = 5; *p* = 0.90). In contrast, GABA levels remained relatively stable across all experimental groups [Cortex, F (3,16) = 2.4, *p* = 0.14, *R* ^2^ = 0.31; 95% CI: −0.43 to 7.65; Hippocampus: F (3,16) = 0.45, *p* = 0.61, *R* ^2^ = 0.08; 95% CI: −9.37 to 3.96; Figure 9B].

Because KA-induced glutamate accumulation stems from excessive release, impaired reuptake, and dysfunctional metabolism (Chang et al., 2024; Lu C. W. et al., 2024; Lu et al., 2026), we analyzed key proteins in the glutamate-glutamine cycle. KA administration significantly downregulated the astrocytic glutamate transporter GLT-1 and the glutamine biosynthetic enzyme GS, while upregulating the glutamine transporters SNAT1/3, neuronal glutaminase, and vesicular packager VGLUT1 compared to the control group [GLT-1: Cortex, F (3,16) = 27.17, *p* < 0.0001, *R* ^2^ = 0.83; 95% CI of decrease: 0.60 to 1.40; Hippocampus: F (3,16) = 63.70, *p* < 0.0001, *R* ^2^ = 0.92; 95% CI of decrease: 0.83 to 1.83; GS: Cortex, F (3,16) = 82.77, *p* < 0.0001, *R* ^2^ = 0.93; 95% CI of decrease: 0.69 to 1.09; Hippocampus: F (3,16) = 37.33, *p* < 0.0001, *R* ^2^ = 0.87; 95% CI of decrease: 0.51 to 1.03; SNAT3: Cortex, F (3,16) = 254.7, *p* < 0.0001, *R* ^2^ = 0.97; 95% CI of increase: 0.74 to 0.96; Hippocampus: F (3,16) = 341.9, *p* < 0.0001, *R* ^2^ = 0.98; 95% CI of increase: 0.74 to 0.82; SNAT1: Cortex, F (3,16) = 290.2, *p* < 0.0001, *R* ^2^ = 0.98; 95% CI of increase: 0.64 to 0.86; Hippocampus: F (3,16) = 116.7, *p* < 0.0001, *R* ^2^ = 0.95; 95% CI of increase: 0.45 to 0.67; Glutaminase: Cortex, F (3,16) = 227.5, *p* < 0.0001, *R* ^2^ = 0.97; 95% CI of increase: 1.49 to 1.95; Hippocampus: F (3,16) = 397.5, *p* < 0.0001, *R* ^2^ = 0.98; 95% CI of increase: 1.42 to 1.72; VGLUT1: Cortex, F (3,16) = 268.8, *p* < 0.0001, *R* ^2^ = 0.98; 95% CI of increase: 0.83 to 1.07; Hippocampus: F (3,16) = 85.87, *p* < 0.0001, *R* ^2^ = 0.94; 95% CI of increase: 0.69 to 1.07; Figure 9C]. This profile reflects increased glutamate synthesis and exocytosis paired with impaired clearance. Pretreatment with PRBS or CBZ effectively reversed these KA-induced alterations (*p* < 0.0001 vs. KA group), with no significant difference between the two treatments. These results suggest that the neuroprotective and anticonvulsant efficacy of PRBS is closely related to the homeostatic regulation of glutamate turnover.

## 4 Discussion

Epilepsy is a prevalent neurological disease, and current pharmacological treatments are often limited by poor efficacy and adverse effects. Recent research has highlighted plant polysaccharides as promising therapeutic candidates (Xie et al., 2022; Lu Y. et al., 2024; Nonato et al., 2024). Our study provides the first evidence that pretreatment with PRBS significantly mitigates KA-induced acute seizures and neuronal damage in rats. This neuroprotection is mediated through three synergistic mechanisms: the suppression of TLR4/NF-κB-driven glial activation and pro-inflammatory cytokine release (IL-1β, IL-6, TNF-α), the stabilization of BBB integrity and CBF, and the maintenance of glutamate homeostasis.

The KA-induced seizure model is widely recognized as a gold standard for the rapid screening of novel antiseizure drugs (Lévesque and Avoli, 2013). Consistent with previous reports (Chang et al., 2022; Hung et al., 2024; Lu et al., 2025), KA administration (15 mg/kg, i.p.) in our study effectively induced tonic convulsions, increased EEG ictal spikes, reduced CBF, and caused substantial neuronal damage in the entorhinal cortex and hippocampus. Notably, oral pretreatment with either PRBS (10 mL/kg) or CBZ (100 mg/kg) significantly mitigated seizure severity, suppressed EEG ictal spikes, maintained CBF, and protected against cortical and hippocampal neuronal damage. In this study, CBZ was used as a positive control because it is a first-line clinical antiepileptic drug (Löscher, 2011). Beyond its classical sodium channel blockade, CBZ has been documented to modulate neurotransmitter release and mitigate acute seizures (Sitges et al., 2007; Hung et al., 2024; Rana et al., 2026), making it a highly relevant clinical benchmark to evaluate the protective efficacy of PRBS against KA-induced excitotoxicity. While a direct dose-equivalency comparison is challenging given that PRBS is a polysaccharide extract and CBZ is a purified small molecule, the robust efficacy of PRBS—which was comparable to the validated therapeutic dose of CBZ—underscores its potent antiseizure and neuroprotective properties, highlighting its substantial pharmacological potential.

Mechanistically, PRBS pretreatment significantly attenuated KA-induced reactive gliosis, as evidenced by reduced densities of GFAP+ astrocytes and OX42+ microglia in the entorhinal cortex and hippocampus. This inhibition was further supported by the downregulation of markers associated with a pro-inflammatory reactive state (C3 for astrocytes and CD86 for microglia) and a concurrent reduction in key pro-inflammatory cytokines (IL-1β, IL-6, and TNF-α). Because glial activation and the ensuing cytokine storm are pivotal drivers of neuronal hyperexcitability and ictogenesis (Vezzani et al., 2013; Sanz and Garcia-Gimeno, 2020; Wolinski et al., 2022), the anti-inflammatory properties of PRBS appear central to its neuroprotective effects. Furthermore, our results indicate that the anti-inflammatory activity of PRBS is closely associated with the modulation of the TLR4/NF-κB signaling pathway. TLR4 activation typically triggers IκB phosphorylation and degradation, allowing NF-κB nuclear translocation, which in turn upregulates pro-inflammatory cytokines and heightens seizure susceptibility (Soltani Khaboushan et al., 2022). Consistent with findings that inhibiting this pathway ameliorates seizures (van Vliet et al., 2018; Liu et al., 2020; Wu et al., 2022; Varmazyar et al., 2025), PRBS significantly reversed the KA-induced elevation of TLR4 expression and the overall activation of the NF-κB signaling pathway. Thus, similar to other plant-derived polysaccharides (Zheng et al., 2020; Xie et al., 2022; Lu Y. et al., 2024), the neuroprotective effects of PRBS against KA-induced acute seizures likely involve the concurrent suppression of the TLR4/NF-κB cascade to mitigate reactive gliosis.

Glial activation and the surge in pro-inflammatory cytokines during seizures disrupt endothelial tight junctions, compromising BBB integrity and thereby exacerbating neuroinflammation and accelerating epilepsy progression (Huang N. et al., 2025; Suleymanova and Karan, 2025). Consequently, stabilizing the BBB is a viable therapeutic strategy against seizures (Greene et al., 2022). In the present study, KA treatment induced significant BBB disruption, marked by enhanced albumin extravasation and the downregulation of tight junction proteins (claudin-5 and occludin) in the cortex and hippocampus—results consistent with prior literature (Noé et al., 2016; Yan et al., 2018; Lu et al., 2026). Importantly, PRBS pretreatment prevented these alterations, preserving BBB integrity. This protective effect is intrinsically linked to the stabilization of CBF, reflecting the preservation of the neurovascular unit (NVU). The reciprocal relationship between BBB disruption and reduced CBF is a hallmark of various neurovascular pathologies (van de Haar et al., 2016; Wong et al., 2019; Gowdy et al., 2026). However, the precise molecular mechanisms by which PRBS modulates this BBB-CBF interplay warrant further investigation.

Neuroinflammation is closely associated with glutamatergic hyperactivity, a core pathological feature of epilepsy (Devinsky et al., 2013; Vezzani and Viviani, 2015; Sumadewi et al., 2023; Onat et al., 2025). We observed a significant increase in glutamate levels in the cortex and hippocampus of KA-treated rats, which was effectively normalized by birch sap pretreatment. To elucidate the basis of this normalization, we quantified key proteins involved in glutamate-glutamine cycle. Physiologically, astrocytic GLT-1 takes up synaptic glutamate, GS converts glutamate to glutamine within astrocytes, SNAT3 exports glutamine from astrocytes, SNAT1 transports glutamine into neurons, glutaminase converts glutamine back to glutamate in neurons, and VGLUT1 packages glutamate into presynaptic vesicles for exocytosis (Shen, 2013; Rose et al., 2018). Following KA exposure, we noted a significant decrease in GLT-1 and GS, accompanied by increased expression of SNAT3, SNAT1, glutaminase, and VGLUT1. These alterations—indicating enhanced neuronal glutamate synthesis and release coupled with impaired astrocytic reuptake—align with previous reports (Chang et al., 2022; Hung et al., 2024; Lu C. W. et al., 2024; Lu et al., 2026). PRBS pretreatment effectively reversed these protein alterations, preserving the physiological release and clearance cycles of glutamate. This restoration of glutamatergic homeostasis is closely associated with the observed reduction in glutamate levels and appears to represent a core component of PRBS’s neuroprotective effects against KA-induced acute seizures.

PRBS is a defined fraction (∼1.29 kDa) comprising fucose, glucose, and fructose (3:5:8) (Huang C. F. et al., 2025). We postulate that its anticonvulsant efficacy stems from these unique physicochemical properties. Although direct BBB penetration was not evaluated, its low molecular weight theoretically favors enhanced systemic absorption and potential brain access compared to typical high-molecular-weight polysaccharides (Chong et al., 2025; Lin et al., 2025). Furthermore, its abundant glucose and fructose constituents could provide critical metabolic support to maintain energy homeostasis during seizure-induced excitotoxicity (Cloix and Hévor, 2009). Importantly, PRBS presents a potential dual anti-inflammatory mechanism: its fucose-rich structure may facilitate direct interaction with immune receptors like TLR4, while its inherent free-radical scavenging capacity mitigates the upstream oxidative stress that triggers TLR4 activation (Yang et al., 2017; Chong et al., 2025). While it remains to be fully elucidated whether PRBS acts primarily as a direct TLR4 antagonist or an upstream antioxidant, these combined characteristics collectively drive its therapeutic potential.

However, our study has several limitations. First, while PRBS modulates TLR4/NF-κB signaling, BBB integrity, CBF, and glutamate homeostasis, the lack of pathway-specific interventions (e.g., targeted inhibitors) precludes confirming direct mechanistic causality versus downstream consequences. In particular, we cannot definitively resolve whether BBB preservation actively drives the reduction in seizures or is a secondary outcome of it. Second, our molecular assessments of BBB integrity and glutamate homeostasis relied heavily on Western blotting. While these provide valuable protein-level insights, future functional validations incorporating tracer extravasation or patch-clamp recordings are essential. Third, the precise active structures and pharmacokinetic profile (including BBB penetrability) of PRBS remain unidentified. Fourth, the KA model typically alters glutamatergic and GABAergic transmission (Friedman et al., 1994; Falcón-Moya et al., 2018; Negrete-Díaz et al., 2022; Zeng et al., 2024). Our unexpected lack of significant GABA alterations may stem from specific temporal assessment windows or limited resolution, warranting high-resolution electrophysiological validation. Finally, the exclusive use of male rats and an acute KA model limits evaluations of sex differences and long-term disease-modifying effects against epileptogenesis, respectively.

**FIGURE 10:**
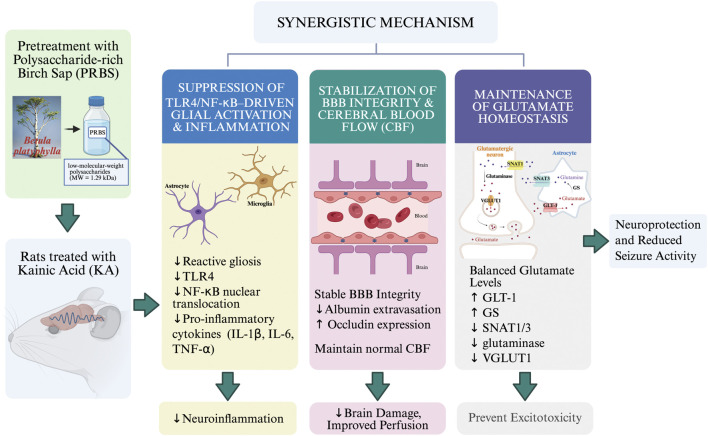
Schematic illustration of the antiseizure and neuroprotective effects of PRBS in the KA-induced rat model. PRBS pretreatment ultimately mitigates KA-induced seizures and neuronal damage in rats by synergistically suppressing TLR4/NF-κB-driven inflammation, stabilizing BBB integrity and CBF, and maintaining glutamate homeostasis (Created with BioRender.com).

In conclusion, this study demonstrates for the first time that pretreatment with PRBS exerts significant antiseizure and neuroprotective effects in a KA-induced rat seizure model. These effects are multi-targeted, involving the concurrent modulation of TLR4/NF-κB-mediated neuroinflammation and reactive gliosis, alongside the preservation of BBB integrity, CBF, and glutamatergic homeostasis (Figure 10). Collectively, these findings highlight PRBS as a highly promising, multi-modal therapeutic candidate for the development of novel pharmacological strategies against acute excitotoxic seizures and related neurological damage.

## Data Availability

The original contributions presented in the study are included in the article/supplementary material, further inquiries can be directed to the corresponding authors.
